# Typical Rhizomatous Clonal Grass *Psammochloa villosa* Changes Resource Allocation During Clonal Expansion to Fit Arid Sandy Habitats

**DOI:** 10.1002/ece3.72432

**Published:** 2025-11-04

**Authors:** Heng Ren, Wen Wang, Wenzhi Zhao, Zhibin He, Jun Du

**Affiliations:** ^1^ Linze Inland River Basin Research Station, Chinese Ecosystem Research Network Northwest Institute of Eco‐Environment and Resources, Chinese Academy of Sciences Lanzhou China; ^2^ Key Laboratory of Ecological Safety and Sustainable Development in Arid Lands Northwest Institute of Eco‐Environment and Resources, Chinese Academy of Sciences Lanzhou China; ^3^ Key Laboratory of Knowledge Computing and Intelligent Decision Lanzhou China

**Keywords:** growth characteristic, mobile dune, morphological traits, patch expansion, *Psammochloa villosa*, rhizomatous plants

## Abstract

Rhizomatous plants forming patchy expansions show high potential for sand fixation in mobile dunes with heterogeneous resources, yet resource allocation strategies among different plant parts and reproductive methods during patch expansion remain poorly understood. This study investigated the growth dynamics and allocation of key morphological traits (plant height, leaf area, and rhizome length) of *Psammochloa villosa* across clonal patches of different sizes in the desert–oasis ecotone of Northwest China. Results revealed that aboveground traits exhibited rapid unimodal growth, peaking in the order of plant height followed by leaf area, whereas rhizomes showed prolonged bimodal growth (approximately 8 months) with delayed peak phases. During clonal expansion from micro to medium patches, asexual clusters decreased the growth rate and increment of leaf area per ramet but markedly increased that of rhizome length, reflecting a shift from photosynthetic to clonal investment. The ratio of belowground to aboveground growth and their allometric relationship both shifted when patch size reached an lg‐transformed area of 2–3, while sexual reproduction occurred exclusively in large patches (lg‐transformed area more than 4). The maximum growth increment of aboveground traits in sexual clusters was more than double that in asexual clusters, indicating a high energy cost associated with sexual reproduction. This was supported by increased aboveground allocation in asexual ramets alongside reduced absolute rhizome growth, despite elevated relative rhizome growth rates. Consequently, sexual reproduction occurs at the expense of rhizome expansion, requiring extensive clonal expansion to accumulate resources in the resource‐limited environment. *P. villosa* thus adapts to arid sandy habitats through stage‐specific resource reallocation during patch expansion, with long‐term spatial occupation via rhizomes preceding the costly sexual reproduction. These insights elucidate the persistence mechanisms of sand‐fixing rhizomatous herbs and highlight the need to quantify threshold patch sizes and ages to enable sexual reproduction across environmental gradients.

## Graphical abstract

Resource allocation of *Psammochloa villosa* changes with clonal expansion in dunes. Growth of plant height, leaf area and rhizome length increase with patch size, and allocation shifts toward aboveground relative to the rhizome at a certain size. Sexual ramets occurs only in sufficiently size at the expense of rhizome growth, highlighting the needs of large spatio‐temporal expansion for sexual reproduction needs in resource‐limited area.

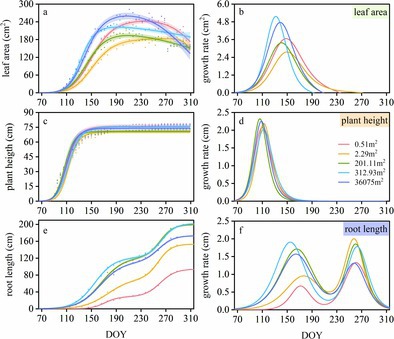

## 1 Introduction

The expansion of mobile dunes and desertification of oasis edges cause movement of the desert‐oasis transitional zone toward the oasis, posing a threat to the stability and maintenance of the oases (Baas and Delobel 2022; Su et al. 2007). It is necessary to implement sand‐fixing measures, such as artificial (mechanical), nature‐based (biological), and hybrid methods, which are tailored to desert ecosystems to slow dune expansion and protect oases (Deng et al. 2024; Wang et al. 2020; Xu et al. 2018). Native plants in sandy environments, due to their good adaptability to wind and drought, exhibit excellent sand‐fixing effects with low water consumption and good persistence (Bel and Ashkenazy 2014; Zhong et al. 2024). In particular, rhizomatous clonal plants can form new ramets and adventitious roots in different directions through horizontal expansion (Guo et al. 2020; Yang et al. 2023), and integrate resources within their clone populations by interconnecting rhizomes (Liu et al. 2016; Yu et al. 2004). These characteristics enable clonal plants to form spatially discrete clonal patches, which are spatially discrete vegetation units dominated by physiologically integrated ramets originating from a single genet that collectively responds to environmental heterogeneity. Through clonal expansion, such patches accumulate and use resources across broader areas, conferring a significant sand‐binding capacity to resource‐heterogeneous mobile and semi‐mobile dunes (Chen et al. 2001; Yang et al. 2023; Zhong et al. 2024). However, current research on self‐sustaining mechanisms (such as natural regeneration and trade‐offs between clonal and sexual reproduction) and resilience of desert ecosystems remain insufficient, constraining our ability to develop artificial vegetation systems with enduring sand‐fixing efficacy.

In arid sandy environments where resources are scarce, vegetation patch size strongly influences soil water and nutrient retention (Su et al. 2007; Wang et al. 2015). Rhizomatous plants typically form clonal patches that spatially expand during growth, redistributing and modifying local resources (such as soil nutrients and water) through root‐rhizome networks (Yu et al. 2004; Zhao et al. 2020). Concurrently, clonal integration enhances nutrient foraging efficiency across patches, enabling ramets in resource‐poor zones to access resources from richer areas as the patch enlarges, thereby mitigating local constraints (Alpert et al. 2003; Liu et al. 2016; Magyar et al. 2007). Owing to growth plasticity, clonal plants at different expansion stages may adjust traits such as phenotypic shape, phenology, and resource allocation in response to habitat changes and growth needs (Cornelissen et al. 2014; Freschet et al. 2018; Liu et al. 2020; Radville et al. 2016; Yang et al. 2023). Furthermore, higher resource availability generally increases clonal plant investment in the more energetically expensive strategy of sexual reproduction compared to vegetative propagation (Bills et al. 2015; Ma et al. 2013; Wang et al. 2018). Studies have indicated that clonal expansion can enhance sexual reproduction efficiency, as manifested by bigger flowers, higher seed‐setting rates and greater spike growth rates relative to reproductive ramets in larger patches (Demetrio and Coelho 2023; Zhou et al. 2015). This is because of the increased number of ramets and rhizomes, which potentially boost sexual reproduction through clonal integration, facilitating resource sharing (such as carbohydrates) from vegetative to reproductive ramets (Demetrio and Coelho 2023; Guo et al. 2020). This integration is critical in resource‐limited deserts, operating along the plant economic spectrum (Reich 2014). Here, clonal growth often represents a conservative strategy (slow return on investment with high tissue longevity), whereas sexual reproduction reflects an acquisitive strategy (high resource investment for rapid dispersal). The trade‐off between these strategies allows for adaptive allocation to either vegetative persistence or generative expansion under stress (Freschet et al. 2018; Yang et al. 2023).

*Psammochloa villosa*, a typical rhizomatous perennial grass, is widely distributed in the deserts of China and Mongolia (Dong and Alaten 1999; Dong et al. 2023). Its adaptability to sand burial, drought, and wind through clonal growth, integration, and morphological adaptability has been studied (Dong and Alaten 1999; Rietkerk et al. 2002; Wang et al. 2011; Yu et al. 2004; Zhong et al. 2024). Rapid clonal expansion and efficient resource integration via rhizomes enable *P. villosa* to survive and expand in deserts with resource heterogeneity (Yu et al. 2004; Zhou et al. 2015). Additionally, *P. villosa* possesses highly adaptive morphological traits: streamlined leaf form reduces wind resistance and mechanical damage, leaf curling minimizes water loss under drought, adventitious root length adjusts to burial depth, and extensive horizontal roots (400–900 cm per ramet) align perpendicularly (approximately 90°) to dune movement. Clonal spacing of about 20 cm forms dense barrier‐like patches, creating a grid structure that reduces wind velocity and promotes sand accumulation (Wang et al. 2011; Zhong et al. 2024). These characteristics make it an important pioneer species on moving dunes, creating distinctive landscapes of interlocking *P. villosa* patches and moving sand patches (Huang et al. 2004; Yu et al. 2004), and a native species with excellent sand‐fixation potential (Chen et al. 2001; Zhong et al. 2024).

However, the dynamics of resource allocation among different plant parts and between reproductive strategies (clonal vs. sexual) throughout the life cycle of *P. villosa*, particularly how these dynamics shift during the critical process of clonal patch expansion, remain poorly understood. This knowledge gap hinders the optimization of its use in sand‐fixing vegetation systems. Therefore, this study focused on *P. villosa* by monitoring key morphological traits (plant height, leaf area, and rhizome length) of both asexual ramets and (when present) sexual ramets over a growth cycle within independent patches of varying sizes. We employed a space‐for‐time approach, using patch size as a surrogate for the expansion process, to investigate resource allocation strategies during patch expansion in an arid sandy habitat. This study aimed to investigate the resource allocation strategy of the rhizomatous clonal plant *P. villosa* during the process of clonal patch expansion in an arid sandy habitat. Specifically, we addressed the following questions: (1) How do the key morphological traits related to photosynthesis (plant height and leaf area) and clonal expansion (rhizome length) grow and interact in response to increasing patch size? (2) How does resource allocation among these traits and between vegetative and reproductive functions shift as patches expand? (3) Is there a critical patch size threshold that enables the initiation of sexual reproduction, and how does achieving this threshold alter the resource allocation patterns? Based on the known plasticity and trade‐offs in clonal plants, we hypothesized that: (H1) Increasing patch size will differentially influence the growth of photosynthetic traits (plant height, leaf area) and the clonal trait (rhizome length), reflecting a trade‐off in resource allocation between above‐ground acquisition and below‐ground expansion/modification, driven by changing internal resource conditions within the expanding patch. (H2) Sexual reproduction will only be initiated after clonal patches reach a critical size threshold enabling sufficient resource accumulation, and patches initiating sexual reproduction will exhibit distinct resource allocation patterns compared to smaller, solely vegetative patches.

## 2 Materials and Methods

### 2.1 Study Area

**FIGURE 1:**
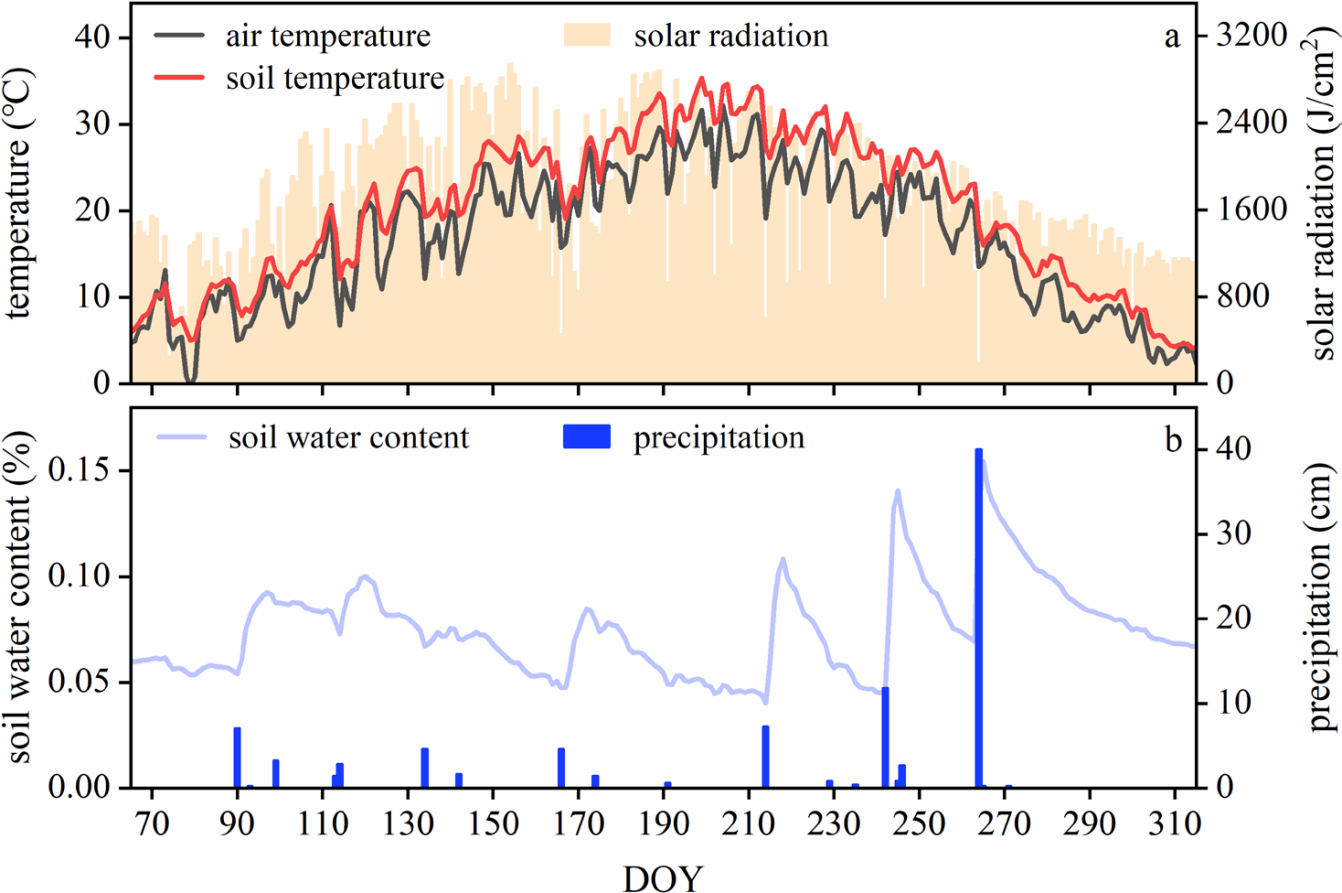
Environmental factors of the study area, including air and soil temperature (a), solar radiation (a), soil water content (b), and precipitation (b).

This study was conducted at the southern edge of the Badain Jaran Desert, one of the four major deserts in northwest China (39°13′19.92″ N, 100°39′00″ E, 1375 m a.s.l.). The study area lies within the transitional zone between desert and oasis and is characterized by a typical continental arid climate with low precipitation, intense radiation and high temperatures (Figure 1). The annual mean temperature in the region is 7.6°C, with a range of 39.1°C to −27.3°C between the highest and lowest recorded temperatures. The annual precipitation averages 117.4 mm, with a potential evaporation of 2390 mm and a drought index of 15.9. The total annual sunshine hours is 3045 h, and solar radiation peaks at 6.1 × 10^5^ J cm^−2^a^−1^ (An et al. 2024). Natural vegetation consists predominantly of xerophytic shrubs (e.g., *Haloxylon ammodendron*, *Calligonum mongolicum*, *Tamarix chinensis* ) and perennial herbs (e.g., *P. villosa* and *Artemisia desertorum*), characterized by a simple community structure, sparse distribution, and an absence of canopy closure (Ren et al. 2022). Observations indicate that the total vegetation cover in the study area was approximately 26%, with a total cover of 22% for the focal plant *P. villosa* (H. Ren, unpublished data), which is the dominant species in the region.

### 2.2 Sample Plot Setting and Monitoring Plant Selection

**FIGURE 2:**
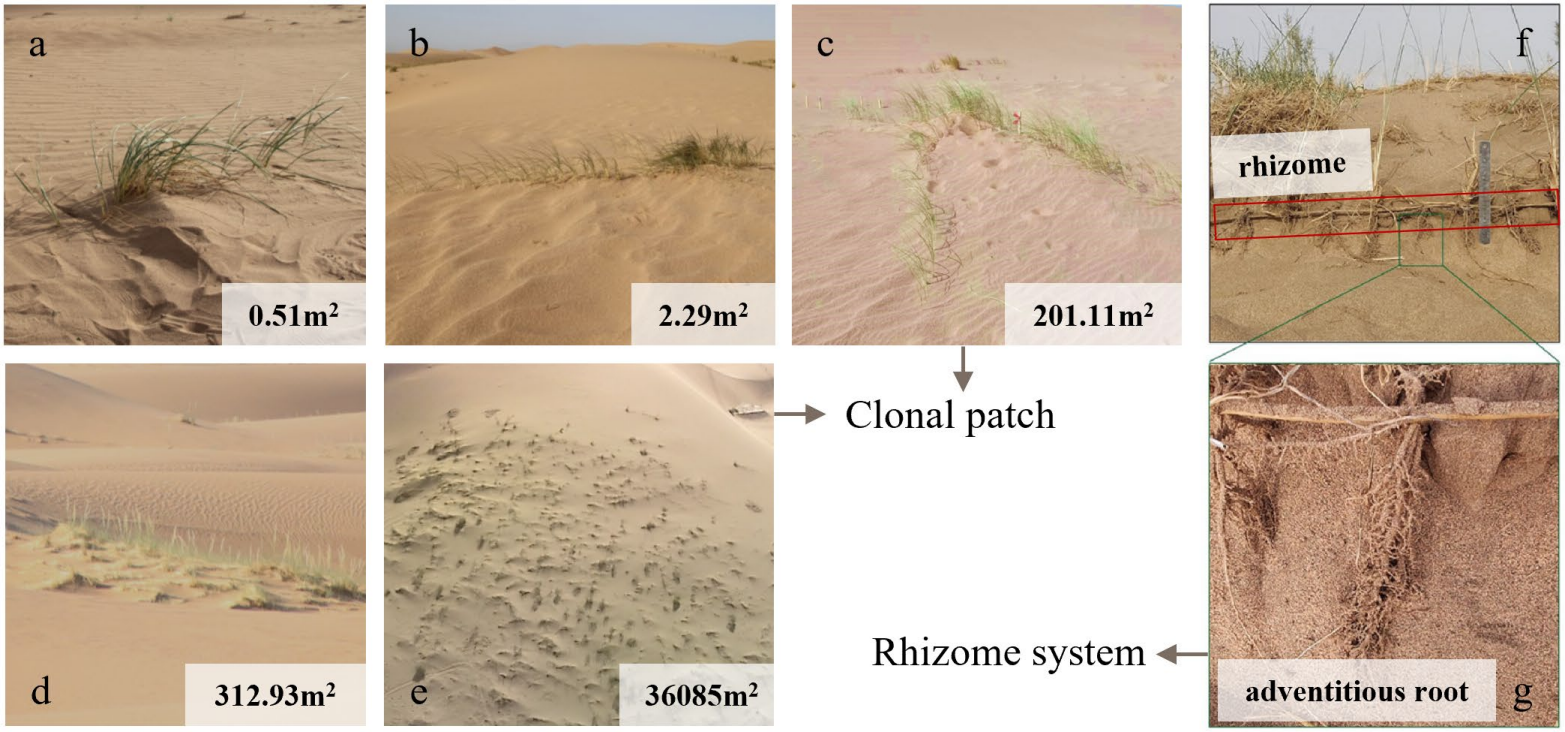
Schematic diagram of *Psammochloa villosa* clonal patches with different sizes and their rhizome system. Images of five sized patches with an area of 0.51 m^2^ (a), 2.29 m^2^ (b), 201.11 m^2^ (c), 312.93 m^2^ (d), and 36,075 m^2^ (e) in this study are shown. The rhizome system image with horizontal rhizome (f) and adventitious root is adapted from Zhong et al. (2024).

To monitor the growth dynamics of the main morphological traits of *P. villosa* as patches expand, five clonal patches with different sizes (0.51, 2.29, 201.11, 312.93, and 36,075 m^2^) were chosen within the mobile dunes of the study area (Figure 2), and all chosen patches were independent of each other. These patches spanned four orders of magnitude in area: micro‐patch (lg area < 0, i.e., 0.51 m^2^), small patch (0 ≤ lg area < 1, i.e., 2.91 m^2^), medium‐sized patches (2 ≤ lg area < 3, represented by smaller patch of 201.11 m^2^ and larger patch of 312.93 m^2^), and large patch (4 ≤ lg area ≤ 5, i.e., 36,075 m^2^), which captures key morphological trait variations during clonal expansion from the settlement stage (micro‐ to medium‐sized patches) to the transition phase from asexual clonal to sexual reproduction (medium‐ to large‐sized patches). To monitor above‐ground morphological traits, clusters (a clump formed by multiple ramets growing from a single point) growing in relatively uniform microhabitats within each patch were selected as monitoring samples. Based on the presence or absence of ramets with flower spikes, the clumps were categorized into sexual or asexual clusters. According to the distribution ratios of sexual and asexual clusters, a total of 26 asexual clusters (including 2, 3, 7, 5, and 8 clusters in the 0.51, 2.29, 201.11, 312.93, and 36,075 m^2^ patches, respectively) and 6 sexual clusters (only found in the 36,075 m^2^ patches within the study area) were selected as monitoring samples. As *P. villosa* is a rhizomatous clonal plant, all ramets within a patch share a common set of rhizomes that can integrate the obtained resources among connected ramets. Therefore, the rhizome samples for monitoring do not distinguish between sexual and asexual ones, but are based on their distribution in patches of different sizes. Ultimately, 2, 3, 7, 5, and 17 rhizomes were selected from the 0.51, 2.29, 201.11, 312.93, and 36,075 m^2^ patches respectively, to monitor the growth dynamics of below‐ground traits.

### 2.3 Main Morphological Traits and Monitoring Methods

In rhizomatous clonal plants, plant height and leaf area directly affect light capture and photosynthesis efficiency, whereas rhizome length is related to population expansion, both of which are crucial for resource acquisition and adaptation to environmental changes (Cornelissen et al. 2014; Sun et al. 2022; Xiao et al. 2007). Therefore, we selected these three morphological traits of *P. villosa* and investigated their growth dynamics during the growing season. According to preliminary investigations, *P. villosa* in the study area returned to green by the end of March. Therefore, plant height and leaf area were monitored once every 5 days, beginning on March 25, 2021. Field investigations for aboveground traits were completed on November 5, 2021, with a total of 45 surveys conducted over the study period. On each survey day, plant height was measured using a ruler (accuracy of 0.1 cm) and recorded as the vertical distance from the highest point to the ground in the natural state for each sample cluster. The measurements were conducted at relatively low wind speeds to minimize the impact of wind. Since a cluster includes multiple ramets, three healthy ramets were selected from a position at similar distances from the tallest ramet in each cluster. The leaf count per ramet was recorded, and the mean leaf number per ramet was calculated. Subsequently, 3–5 intact and disease‐free leaves of each ramet were selected based on representative morphology and size. The area of these leaves was measured using a Yaxin‐1241 portable leaf area instrument (accuracy of 0.1 mm^2^). The mean leaf area per leaf for the cluster was derived by averaging measurements from all sampled leaves. The mean leaf area per ramet was then calculated as mean leaf area per leaf pulsing mean leaf number per ramet and served as the cluster‐level metric for subsequent analyses of leaf‐area‐related traits.

**FIGURE 3:**
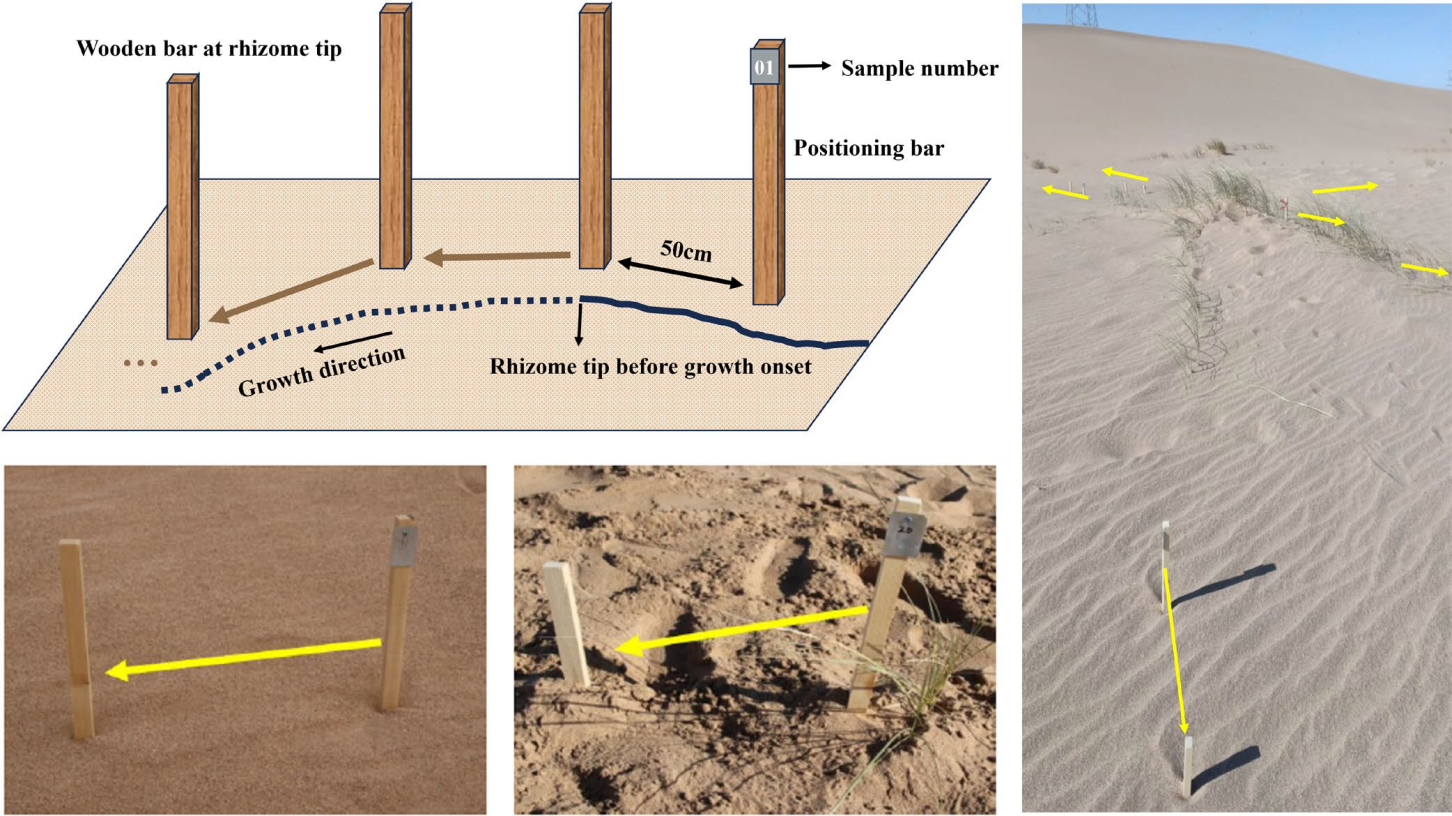
The “excavation and trace root” method of rhizome length monitoring. We selected target rhizomes for investigation by gently removing the surface soil to expose them. During the initial survey, a 60‐cm tall wooden bar was inserted parallel to the rhizome tip. Simultaneously, a positioning bar equipped with a numbered iron tag was inserted 50 cm away from the rhizome tip in the direction opposite to the rhizome's growth. The soil was then gently backfilled. These two bars effectively marked the position of the rhizome tip and its growth direction. In each subsequent survey, the positioning bar was relocated to the position of the previous tip. Starting from this point, surface soil was gently removed along the growth direction to expose the rhizome, and another bar was inserted to mark the current tip position. After marking, the soil was backfilled as quickly and gently as possible. The distance between the two bars was measured using a ruler with an accuracy of 0.1 cm and recorded as the growth increment for that survey period.

Because the growth of the rhizome commenced at the end of February, according to the survey, monitoring of the rhizome length of the selected samples began on February 25, 2021. Since continuous monitoring of below‐ground organs is relatively difficult, after trying various methods, this study achieved non‐destructive continuous monitoring of rhizome length utilizing the “excavation and trace root” method (Figure 3). Target rhizomes were selected for investigation by gently removing the surface soil to expose them. During the initial survey, a 60‐cm tall wooden bar was inserted parallel to the rhizome tip. Simultaneously, a positioning bar equipped with a numbered iron tag was inserted 50 cm away from the rhizome tip in the direction opposite to that of rhizome growth. The soil was then gently backfilled. These two bars effectively marked the position of the rhizome tip and its growth direction. In each subsequent survey, the positioning bar was relocated to the position of the previous tip. Starting from this point, surface soil was gently removed along the growth direction to expose the rhizome, and another bar was inserted to mark the current tip position. After marking, the soil was backfilled as quickly and gently as possible. The distance between the two bars was measured using a ruler with an accuracy of 0.1 cm and recorded as the growth increment for that survey period. Since *P. villosa* rhizomes grow horizontally, merely removing the surface soil exposes them without requiring extraction from the ground, thereby causing minimal damage. Furthermore, by only excavating the newly grown rhizome segment since the last monitoring event, the disturbance to the rhizome from soil removal was minimized. To further mitigate the disturbance to rhizome growth caused by excavation, the monitoring frequency was regulated to once every 10 days (i.e., aboveground traits were assessed alone in a specific survey, with both above‐ and belowground traits measured simultaneously in the subsequent survey). Field investigations for rhizome length were completed on December 15, 2021, with a total of 30 surveys conducted over the study period.

### 2.4 Acquisition of Growth Characteristic Parameters

Since *P. villosa* is a perennial herb, its above‐ground parts regrow after withering each year; therefore, the data for plant height and leaf area start from zero, with each measurement representing the growth increment at each monitoring time. Otherwise, the growth of the rhizome is based on the original foundation, so the value measured for the first time was used as the initial value, and the subsequent growth increment was calculated by subtracting the initial value from each measurement. All calendar dates were subsequently converted to their corresponding days of the year (DOY), a numerical value representing sequential days within 1 year (e.g., January 1 = 1; January 2 = 2, etc.). Higher DOY values indicate later dates in the annual cycle. Suitable growth functions were selected and the growth increments of all samples were fitted and differentiated separately (Table 1). The growth characteristic parameters of each monitored sample of ramets and rhizomes were extracted from the fitted and derivative curves. The extracted parameters of growth characteristics included: maximum growth increment (*W*_max_, the maximum value of the fitted curve which represents the total increments in plant height, leaf area, or rhizome length), maximum growth rate (*V*_max_, the maximum value of the derivative), onset date of growth (*D*_*o*_, the date when the growth increment first changed), date when rapid growth began (*D*_*r*_), date when the maximum growth rate was reached (*D*_vmax_), onset date of stable growth (*D*_st_), and date of growth termination (*D*_*t*_, the date when the maximum growth increment was reached). The slow growth period (*D*_sl_, number of days between *D*_*r*_ and *D*_*o*_), rapid growth period (GD_*r*_, number of days between *D*_st_ and *D*_*r*_), stable growth period (GD_st_, number of days between *D*_*t*_ and *D*_st_), and total growth period (GD, the number of days between *D*_*t*_ and *D*_*o*_) were also calculated. The mean growth rate (*V*_mean_, i.e., the growth increment per day) was also calculated by *W*_max_/GD. In addition, the mean growth increment data of all samples belonging to the population or each size category were used for growth curve fitting and differentiation of the fitted curves to visualize the growth dynamics and growth rate changes of the main morphological traits for the population or each size category over one growing season (Table 1).

**TABLE 1:** Growth functions used for trait fitting.

Traits	Models	Function, parameters, and the range of adjusted *R* ^2^
Plant height	Logistic	Function: $y=A_{2}+\frac{A_{1}-A_{2}}{1+{\left(\frac{x}{x_{0}}\right)}^{p}}$
		Parameters (4): *A*_1_ = initial value, *A*_2_ = final value, *x*_0_ = center, *p* = power
		Adjusted *R* ^2^: 0.966–0.997 (*p* < 0.01)
Leaf area	BiHill	Function: $y=\frac{P_{m}}{\left[1+{\left(\frac{K_{a}}{x}\right)}^{H_{a}}\right]\left[1+{\left(\frac{x}{K_{i}}\right)}^{H_{i}}\right]}$
		Parameters (5): *P*_*m*_ = maximum, *K*_*a*_ = half‐maximal activating, *K*_*i*_ = half‐maximal inhibitory, *H*_*a*_ = activation Hill coefficient, *H*_*i*_ = inhibitory Hill coefficient
		Adjusted *R* ^2^: 0.943–0.994 (*p* < 0.01)
Rhizome length	BiDoseResp	Function: $y=A_{1}+\left(A_{2}-A_{1}\right)\left[\frac{P}{1+10^{\left({\mathrm{LOG}}_{10}01-x\right)h1}}+\frac{P}{1+10^{\left({\mathrm{LOG}}_{10}02-x\right)h2}}\right]$
		Parameters (7): *A*_1_ = Bottom, *A*_2_ = Top, LOG_*x*_01 = 1st EC50, LOG_*x*_02 = 2nd EC50, *h*1 = slope1, *h*2 = slope2, *P* = proportion
		Adjusted *R* ^2^: 0.992–0.999 (*p* < 0.01)

### 2.5 Statistical Analysis

To compare the differences in the growth characteristics of various morphological traits at the population level, all monitored samples were used as replicates to analyze the differences in each growth characteristic parameter. Subsequently, for all growth characteristic parameters of each morphological trait, the differences between patches were analyzed using samples from different size categories. Because the large patch (36,075 m^2^) contained both sexual and asexual clusters, and there were obvious differences in their above‐ground morphological traits, to ensure comparability between different patches, only the data of asexual clusters from the large patch were used for comparison with other patches when comparing above‐ground morphological traits. Owing to the limitation of single‐patch representation per size category (*N* = 1 patch per size class), formal statistical comparisons between patches were precluded. Instead, we performed descriptive analyses by applying *β*‐spline curve fitting to visualize trait trends along the logarithmic area gradient (lg‐transformed patch area). Traits were measured at the cluster level, with patch‐level means serving as input for spline fitting. *β*‐spline fitting is a flexible smoothing technique that captures non‐linear trends in data without assuming a fixed functional form. It was particularly suitable here given the exploratory nature of our patch‐size analysis and the need to visualize continuous trait variation across a logarithmic‐area gradient.

Simultaneously, the growth characteristic parameters of the two types of clusters in the large patch were compared to explore the differences in the growth characteristics of their above‐ground morphological traits. Their differences in growth characteristic parameters were analyzed using a one‐way analysis of variance (ANOVA). Prior to analysis, homogeneity of variance was verified. For data meeting the homogeneity assumption, a standard ANOVA *F*‐test was performed. Otherwise, Welch's progressive *F* tests were implemented. The above analysis was performed using the stats package in R 4.5.0 (R Core Team 2025); *p* ≤ 0.05 was considered to indicate a significant difference, and 0.05 < *p* < 0.10 was considered to indicate marginal significance.

The differences in growth characteristics between different traits can to some extent reflect the allocation results of plants (Bai et al. 2020; Cornelissen et al. 2014). Therefore, to investigate resource allocation patterns of morphological traits among patches, we calculated and compared ratios of trait growth increments among patches based on mean trait values per patch, including aboveground ratio of leaf area to plant height (LA‐PH) and belowground‐aboveground ratios of rhizome length to leaf area (RL‐LA) and rhizome length to plant height (RL‐PH). Furthermore, to examine patch differences in relative growth relationships between traits, we applied the allometric growth model (*y* = *ax* ^*b*^): mean trait values per patch were log‐transformed (lg *y* = lg *a* + *b* lg *x*), followed by Standardized Major Axis regression (SMA) to establish growth relationships for trait pairs (LA‐PH, RL‐LA, RL‐PH). Here, independent variable *x* represented leaf area or rhizome length, dependent variable *y* denoted plant height or leaf area under identical conditions, and slope *b* constituted the allometric index, with higher *b* values indicating greater growth rates of trait *y* relative to *x*. We also applied *β*‐spline curve fitting to visualize slope trends along the logarithmic area gradient (lg‐transformed patch area). All analyses were conducted in R 4.5.0 using the smatr package (v3.4.8; Warton et al. 2012). Field observations revealed that aboveground plant height and leaf area of *P. villosa* stabilized after mid‐July, whereas rhizome length continued increasing until early November, suggesting stage‐specific resource allocation. Consequently, we focused on trait growth relationships prior to mid‐July.

## 3 Results

### 3.1 Growth Characteristics of the Main Morphological Traits of the Population

**FIGURE 4:**
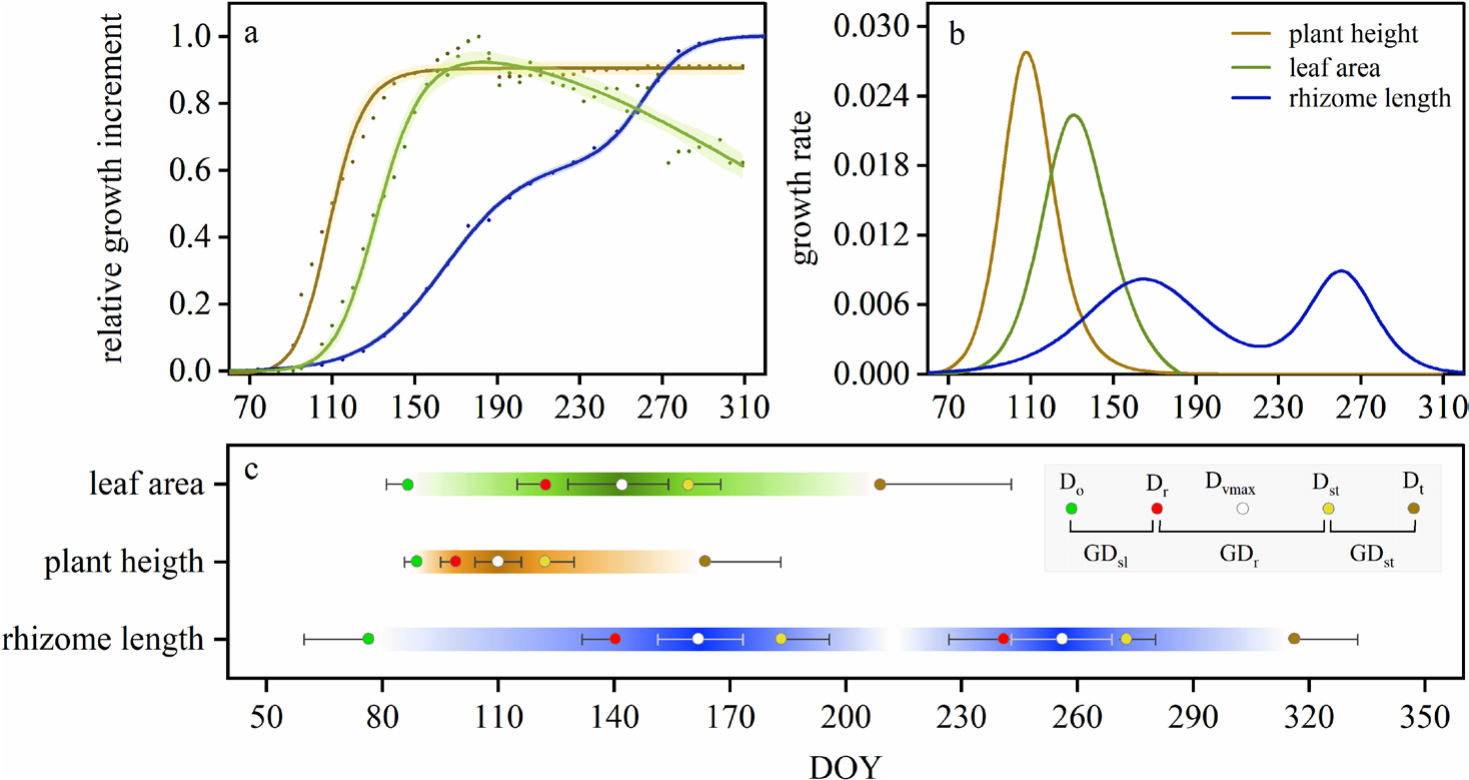
Growth characteristics of three morphological traits of the population, including (a) relative growth increment, (b) growth rate, and (c) growth phenology. The average of the growth increment data for all samples in the population was standardized, followed by fitting a growth curve which was shown in (a) and taking the derivative of the fitted curves which were shown in (b), to intuitively display the growth characteristics of each trait. The mean of the phenological growth parameters extracted after fitting each sample in the population was represented in (c). *D*_*o*_, onset date of growth; *D*_*r*_, the date when rapid growth began; *D*_sl_, slow growth period; *D*_st_, onset date of stable growth; *D*_*t*_, the date of growth termination; *D*_vmax_, the date when the maximum growth rate was reached; GD, total growth period; GD_*r*_, rapid growth period; GD_st_, stable growth period.

There were significant differences in phenological characteristics and growth patterns among the three morphological traits of *P. villosa*. For the above‐ground parts, plant height and leaf area exhibited a unimodal growth pattern that lasted approximately two and a half months (from the end of March to mid‐June) in the early growing season (Figure 4a–c). Both traits began to grow at a similar time, while the time plant height entered the rapid growth period (*D*_*r*_), reached the maximum growth rate (*D*_vmax_), and entered a stable growth period (*D*_st_) was significantly earlier (*p* < 0.001) (Figure 4c, Table 2). The total growth period (GD) of plant height was significantly shorter than that of leaf area (*p* < 0.001), with the stable growth period (GD_st_) being significantly longer than that of the leaf area (*p* < 0.001) (Figure 4c, Table 2). For the below‐ground part, rhizome growth began approximately 10 days earlier (*p* < 0.001), and terminated approximately 4 months later (*p* < 0.001) than the above‐ground traits, resulting in a total growth period that was nearly two times longer than that of the latter (*p* < 0.001), lasting approximately 8 months (Figure 4c, Table 2). Furthermore, the rhizome length exhibited a bimodal growth pattern, with its first peak occurring in mid‐June, significantly later than that of the above‐ground traits (*p* < 0.001); and its initial rapid growth period ended in early July, after which growth slowed down until rapid growth resumed again at the beginning of September (Figure 4b,c, Table 2). Collectively, the prolonged rhizome growth period with bimodal rhythm versus ephemeral unimodal aboveground development reflects a temporal niche‐partitioning strategy among different traits.

**TABLE 2:** Comparison of phenology of growth for different morphological traits.

Phenological parameters	Trait			ANOVA	
	Leaf area	Plant height	Rhizome length	*F*	*p* <
*D*_*o*_ (DOY)	86 ± 5a	88 ± 2a	77 ± 17b	8.102	0.001
*D*_*r*_ (DOY)	122 ± 7b	98 ± 3c	141 ± 9a	346.98	0.001
*D*_vmax_ (DOY)	141 ± 13b	110 ± 6c	162 ± 11a	435.62	0.001
*D*_st_ (DOY)	161 ± 7b	121 ± 9c	182 ± 13a	276.24	0.001
*D*_*t*_ (DOY)	209 ± 34b	163 ± 20c	316 ± 17a	594.95	0.001
GD_sl_ (day)	36 ± 8b	11 ± 4c	64 ± 16a	286.52	0.001
GD_*r*_ (day)	39 ± 15b	23 ± 6b	41 ± 9a	63.08	0.001
GD_st_ (day)	48 ± 21c	42 ± 15b	60 ± 14a	201.02	0.001
GD (day)	123 ± 35b	75 ± 21c	239 ± 28a	381.29	0.001

*Note:* Values are presented as mean ± SD Values within the same parameter followed by the same lowercase letters are not significantly different among traits based on the LSD test or Games‐Howell test at the 0.05 level.

Abbreviations: *D*_*o*_, onset date of growth; *D*_*r*_, the date when rapid growth began; *D*_sl_, slow growth period; *D*_st_, onset date of stable growth; *D*_*t*_, the date of growth termination; *D*_vmax_, the date when the maximum growth rate was reached; GD, total growth period; GD_*r*_, rapid growth period; GD_st_, stable growth period.

### 3.2 Changes of Growth Characteristics of the Main Morphological Traits During Clonal Expansion

**FIGURE 5:**
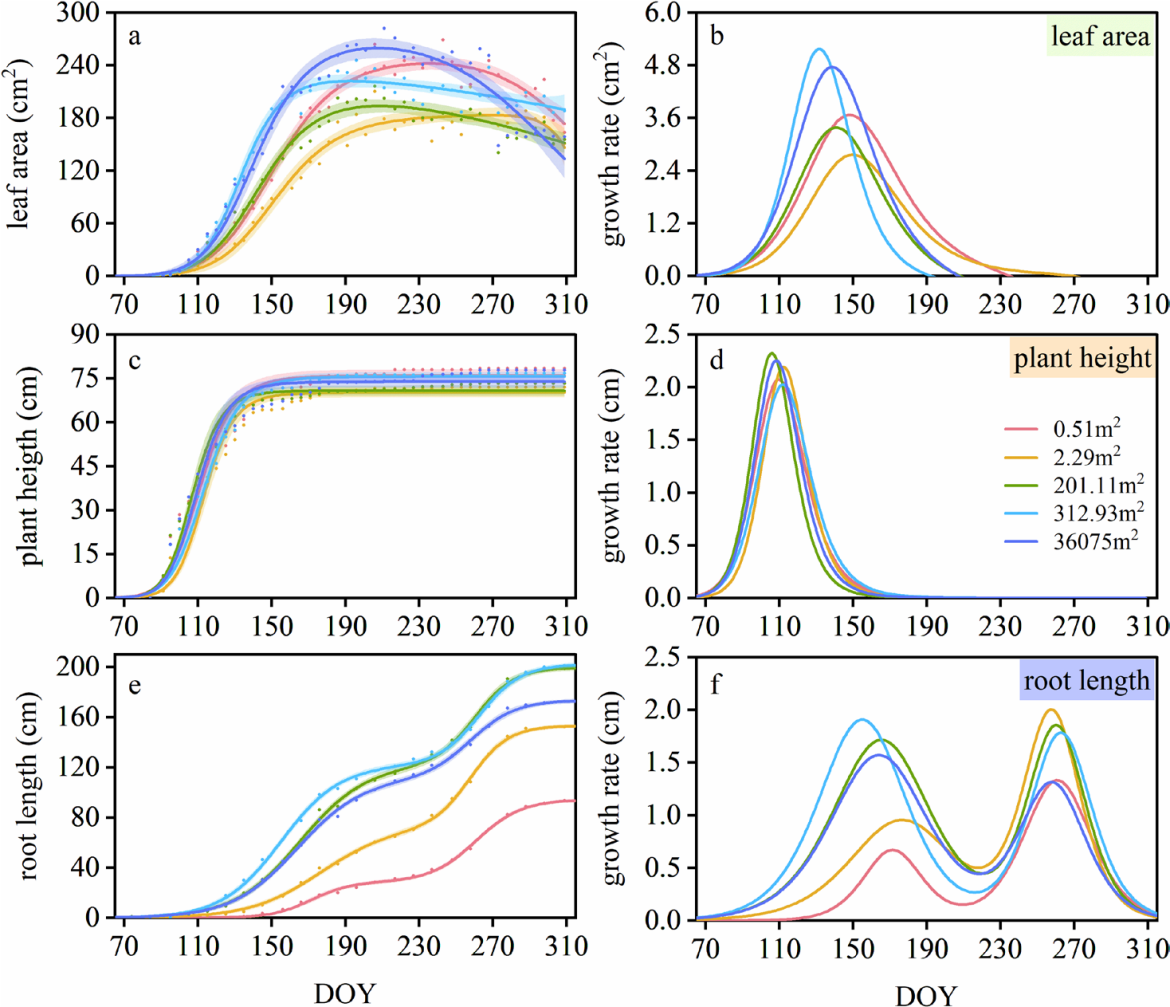
Growth characteristics of leaf area, plant height, and rhizome length of different patch sizes, including relative growth increment (a, c, e), and growth rate (b, d, f). The average of the growth increment data for samples belonging to each patch size was standardized, followed by fitting growth curves which were shown in (a, c, e) and taking the derivative of the fitted curves which were shown in (b, d, f), to intuitively display the growth characteristics of each patch size.

**FIGURE 6:**
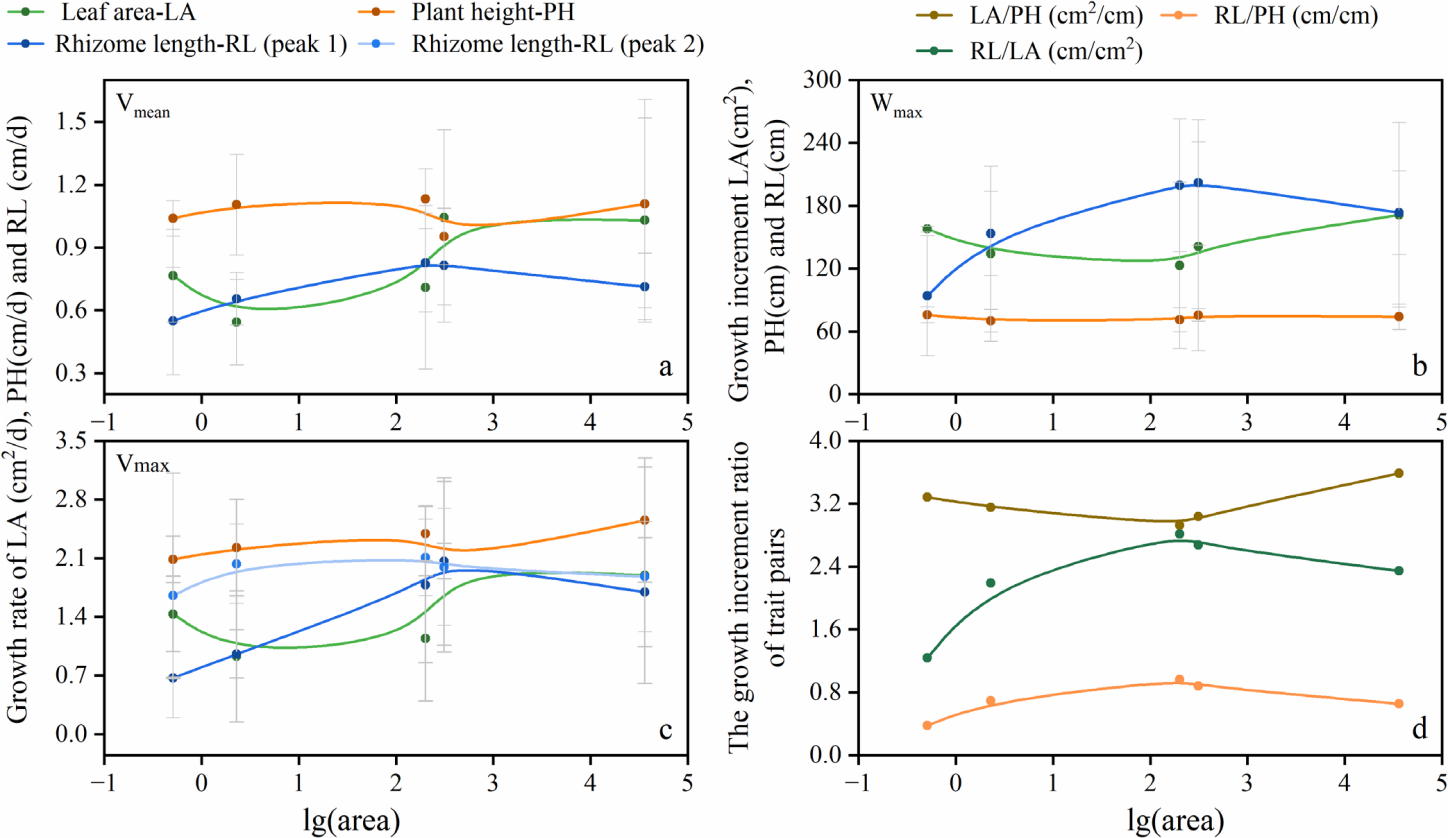
Changes in growth rate (a: mean rate, c: max rate), growth increment (b), and trait pair ratios of growth increment (d) during patch expansion. Area of patches was lg‐transformed. The *β*‐spline curve was fitted to visualize trait trends along the logarithmic area gradient (lg‐transformed patch area). Traits were measured at the cluster level, with patch‐level means (plot in images) serving as input for spline fitting. Error bars represent ±1 SD.

Asexual clusters occurred consistently across all patches. Both its growth rate and growth increment of leaf area (mean leaf area per ramet, hereafter) exhibited an initial decline followed by an increase during clonal expansion, with the mean growth rate manifesting its minimum in small patches, whereas the lowest growth increment occurred in medium‐sized patches (Figures 5a,b and 6a–c). Neither the growth rate nor the growth increment of its plant height exhibited obvious trends along the patch size gradient (Figure 6a–c, Table 3). The growth rate and growth increment of rhizome length (mean patch‐level rhizome length, irrespective of reproductive strategy, hereafter) showed an initial increase followed by a decline, and their peak values within medium‐sized patches were approximately 3‐fold (about 100 cm^2^ in maximum growth increment and 1 cm^2^/day in maximum growth rate) greater than those minima observed in micro‐patches (Figure 6a–c, Table 3). Furthermore, during patch expansion, the aboveground leaf area‐to‐plant height ratio exhibited a unimodal pattern, while the belowground‐to‐aboveground ratios (rhizome length to leaf area or plant height) both displayed hump‐shaped trends (Figure 6d, Table 3). Concurrently, medium‐sized patches demonstrated the lowest growth allocation to leaf area relative to plant height, yet the highest aboveground‐to‐belowground biomass partitioning (Figure 6d, Table 3).

**TABLE 3:** Growth characteristics comparison of leaf area, plant height and rhizome length among different patch size.

Traits	Growth parameters	Patch size				
		0.51 m^2^	2.29 m^2^	201.11 m^2^	312.93 m^2^	36,075 m^2^
Leaf area	*V*_mean_ (cm^2^/day)	1.7 ± 0.4	1.3 ± 0.4	1.6 ± 0.8	2.3 ± 0.8	2.3 ± 1.0
	*V*_max_ (cm^2^/day)	4.1 ± 1.0	3.0 ± 1.7	3.5 ± 1.6	5.3 ± 2.2	5.1 ± 2.8
	*W*_max_ (cm^2^)	249 ± 3	221 ± 100	208 ± 95	229 ± 120	266 ± 106
	*D*_*o*_ (DOY)	91 ± 8	88 ± 2	85 ± 5	88 ± 5	84 ± 5
	*D*_*r*_ (DOY)	130 ± 13	131 ± 7	124 ± 6	117 ± 4	122 ± 5
	*D*_vmax_ (DOY)	151 ± 18	158 ± 14	147 ± 15	132 ± 6	142 ± 9
	*D*_st_ (DOY)	172 ± 22	185 ± 19	171 ± 24	147 ± 9	163 ± 15
	*D*_*t*_ (DOY)	239 ± 44	258 ± 53	224 ± 40	186 ± 18	205 ± 17
	GD_sl_ (day)	39 ± 6	43 ± 9	38 ± 8	28 ± 8	37 ± 8
	GD_*r*_ (day)	42 ± 8	54 ± 13	47 ± 19	30 ± 6	41 ± 12
	GD_st_ (day)	68 ± 22	73 ± 42	53 ± 24	39 ± 13	42 ± 16
	GD (day)	149 ± 36	170 ± 54	139 ± 40	98 ± 21	120 ± 21
Plant height	*V*_mean_ (cm/day)	1.0 ± 0.1	1.1 ± 0.2	1.1 ± 0.1	1.0 ± 0.1	1.1 ± 0.5
	*V*_max_ (cm/day)	2.1 ± 0.3	2.2 ± 0.6	2.4 ± 0.3	2.1 ± 0.2	2.6 ± 0.7
	*W*_max_ (cm)	76 ± 8	70 ± 11	71 ± 11	76 ± 6	74 ± 12
	*D*_*o*_ (DOY)	87 ± 1	91 ± 4	87 ± 2	88 ± 2	88 ± 1
	*D*_*r*_ (DOY)	98 ± 1	100 ± 3	96 ± 2	99 ± 2	97 ± 3
	*D*_vmax_ (DOY)	110 ± 1	111 ± 3	106 ± 3	111 ± 4	107 ± 6
	*D*_st_ (DOY)	122 ± 1	121 ± 4	116 ± 5	123 ± 5	118 ± 9
	*D*_*t*_ (DOY)	160 ± 0	155 ± 6	151 ± 10	168 ± 9	167 ± 31
	GD_sl_ (day)	11 ± 1	9 ± 1	9 ± 2	10 ± 1	9 ± 3
	GD_*r*_ (day)	25 ± 1	21 ± 3	20 ± 4	25 ± 3	20 ± 6
	GD_st_ (day)	38 ± 1	34 ± 3	35 ± 6	45 ± 6	49 ± 25
	GD (day)	73 ± 1	64 ± 6	63 ± 12	80 ± 7	79 ± 31
Rhizome length	*V*_mean_ (cm/day)	0.5 ± 0.3	0.7 ± 0.1	0.8 ± 0.2	0.8 ± 0.3	0.7 ± 0.2
	*V*_max_ (cm/day)	0.7 ± 0.0	1.0 ± 0.3	1.8 ± 0.9	2.1 ± 1.0	1.7 ± 0.7
	*V*_max‐2_ (cm/day)	1.7 ± 1.5	2 ± 0.5	2.1 ± 0.5	2 ± 0.7	1.9 ± 0.7
	*W*_max_ (cm)	94 ± 57	153 ± 40	200 ± 63	202 ± 60	173 ± 40
	*D*_*o*_ (DOY)	129 ± 1	84 ± 21	72 ± 8	72 ± 8	73 ± 12
	*D*_*r*_ (DOY)	157 ± 4	153 ± 4	141 ± 5	135 ± 10	140 ± 8
	*D*_vmax_ (DOY)	171 ± 6	177 ± 1	164 ± 7	156 ± 15	161 ± 9
	*D*_st_ (DOY)	185 ± 8	198 ± 2	185 ± 9	176 ± 19	181 ± 12
	*D*_*r*‐2_ (DOY)	238 ± 23	245 ± 1	247 ± 12	249 ± 13	241 ± 14
	*D*_vmax‐2_ (DOY)	251 ± 21	258 ± 2	259 ± 9	262 ± 8	256 ± 11
	*D*_st‐2_ (DOY)	265 ± 20	272 ± 3	273 ± 5	277 ± 3	271 ± 8
	*D*_*t*_ (DOY)	294 ± 26	316 ± 11	312 ± 14	321 ± 14	317 ± 19
	GD_sl_ (day)	28 ± 6	69 ± 17	68 ± 10	64 ± 15	67 ± 15
	GD_*r*_ (day)	28 ± 4	45 ± 5	45 ± 7	41 ± 10	41 ± 9
	GD_sl‐2_ (day)	54 ± 15	47 ± 2	61 ± 14	73 ± 17	61 ± 9
	GD_*r*‐2_ (day)	27 ± 3	27 ± 3	26 ± 9	28 ± 11	30 ± 10
	GD_st_ (day)	29 ± 6	44 ± 10	40 ± 16	45 ± 17	71 ± 45
	GD (day)	165 ± 28	232 ± 19	240 ± 18	250 ± 20	244 ± 24

*Note:* Values are presented as mean ± SD. *V*_max‐2_, *D*_*r*‐2_, *D*_vmax‐2_, *D*_st‐2_, GD_sl‐2_, and GD_*r*‐2_ are the parameters of rhizome length during the late growth period; other abbreviations for each parameter are the same as those in Table 1.

Abbreviations: *V*_max_, maximum growth rate; *V*_mean_, mean growth rate; *W*_max_, maximum growth increment.

**FIGURE 7:**
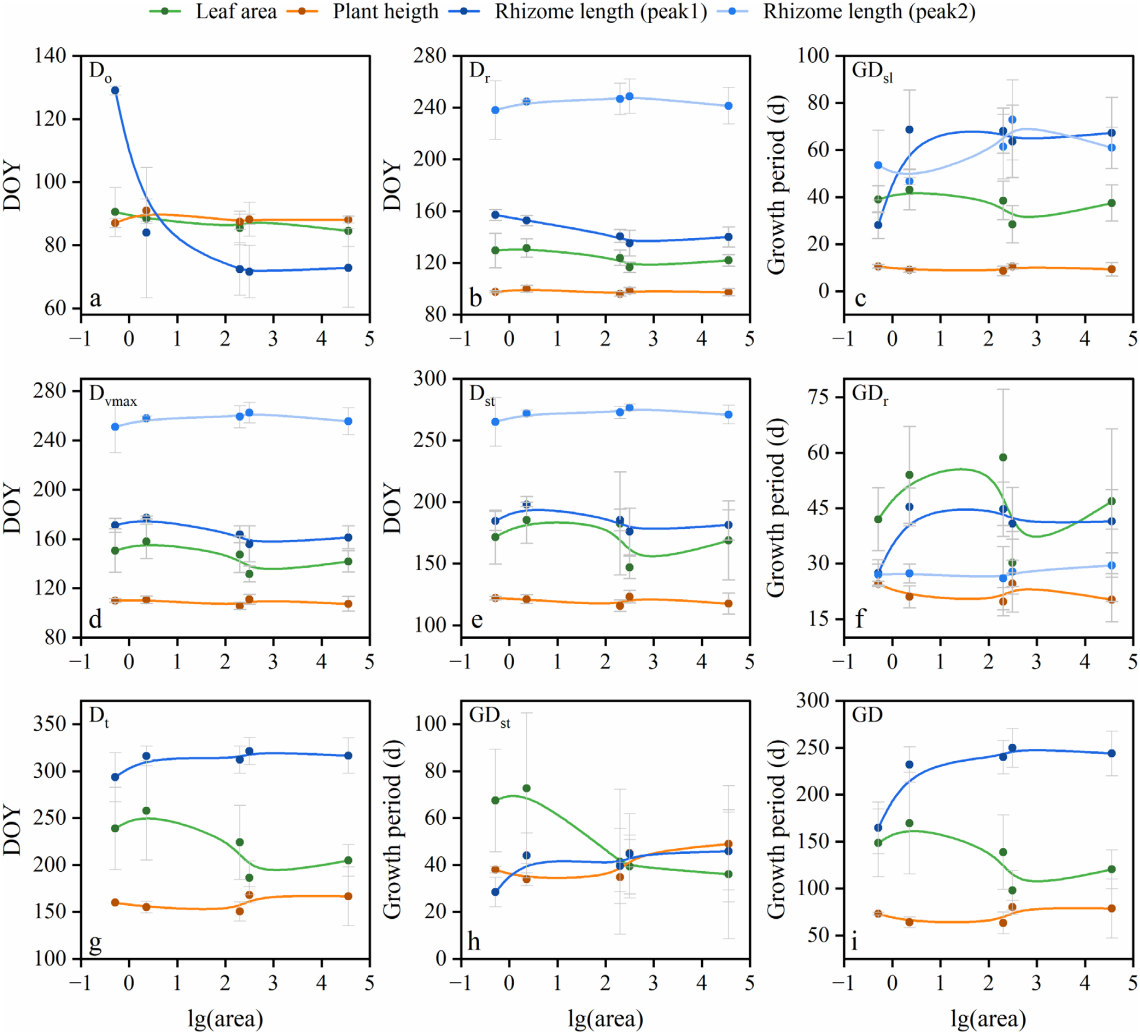
Changes in phenological metrics during patch expansion. Area of patches was lg‐transformed. The *β*‐spline curve was fitted to visualize trait trends along the logarithmic area gradient (lg‐transformed patch area). Traits were measured at the cluster level, with patch‐level means (plot in images) serving as input for spline fitting. Error bars represent ±1 SD. *D*_*o*_: onset date of growth (a); *D*_*r*_: the date when rapid growth began (b); *D*_vmax_: the date when the maximum growth rate was reached (d); *D*_st_: onset date of stable growth (e); *D*_*t*_: the date of growth termination (g); GD_sl_: slow growth period (c); GD_*r*_: rapid growth period (f); GD_st_: stable growth period (h); GD: total growth period (i).

Phenologically, the initial growth onset (*D*_*o*_) of aboveground traits (plant height and leaf area) remained invariant across patch expansion, whereas rhizome initiation advanced obviously (Figure 7a, Table 3). Consequently, aboveground onset preceded belowground initiation by approximately 40 days in the micro‐patch, narrowing to about a 10‐day precedence in medium and large patches (Figure 7a, Table 3). Leaf area growth termination (*D*_*t*_) advanced progressively with expansion, shortening the total growth period (GD); its rapid growth period (GD_r_) extended during the micro‐to‐medium transition, contracted in medium patches, then recovered in the large patch (Figure 7f,g,i, Table 3). Plant height exhibited no obvious phenological shifts. For rhizomes, the first slow growth period (GD_sl_), first rapid growth period (GD_*r*_), and GD all increased during the micro‐to‐medium expansion before stabilizing (Figure 7c,f,i, Table 3). From plant height to leaf area to rhizome length, growth termination was progressively delayed and the total growth period was progressively extended across all patches (Figure 7g,i, Table 3). Specifically in the micro‐patch, GD of rhizome length approximated that of leaf area while exceeding plant height by more than 2‐fold (Figure 7i, Table 3). Furthermore, patch expansion induced prolonged GD of rhizome length but shortened that of leaf area, resulting in GD of rhizome length exceeding both aboveground traits by more than two‐fold (> 100 days) in medium and large patches (Figure 7i, Table 3).

### 3.3 Difference of Above‐Ground Growth Characteristics Between Sexual and Asexual Clusters

**FIGURE 8:**
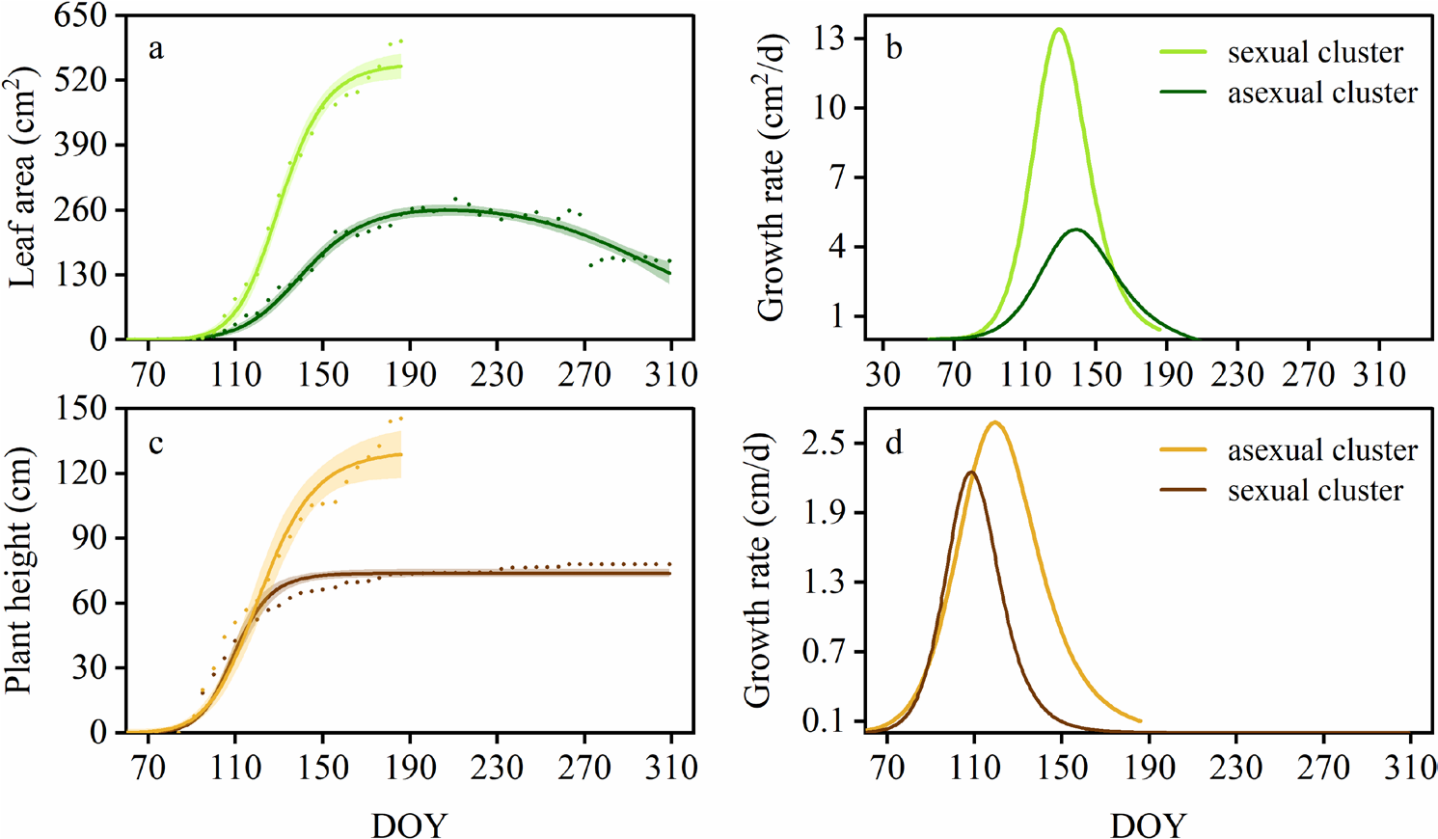
Growth characteristics of leaf area and plant height of asexual and sexual clusters, including relative growth increment (a, c), and growth rate (b, d). The average of the growth increment data for samples belonging to asexual and sexual clusters was standardized, followed by fitting growth curves which were shown in (a, c) and taking the derivative of the fitted curves which were shown in (b, d), to intuitively display the growth characteristics of each patch size.

**FIGURE 9:**
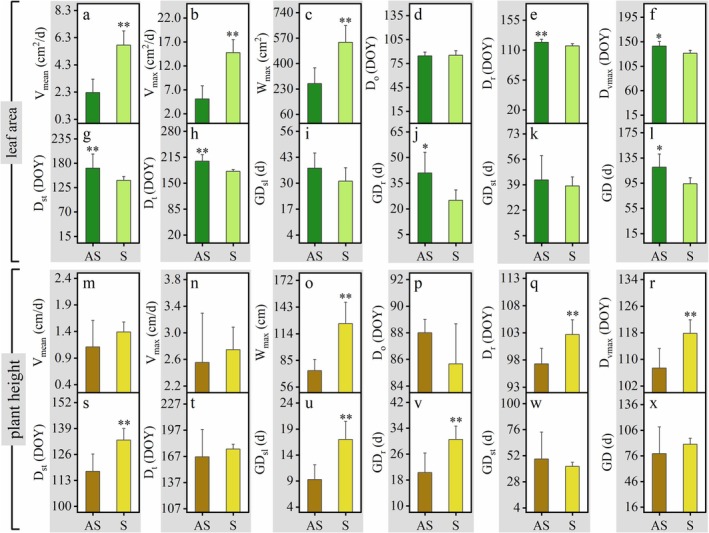
Growth characteristics comparison of leaf area (a–l) and plant height (m–x) between asexual and sexual clusters. Asexual cluster abbreviated as AS in the figure, is denoted by dark bars; the sexual cluster, abbreviated as S, is denoted by light bars. *D*_*o*_, onset date of growth; *D*_*r*_: the date when rapid growth began; *D*_sl_, slow growth period; *D*_st_, onset date of stable growth; *D*_*t*_, the date of growth termination; *D*_vmax_, the date when the maximum growth rate was reached; GD, total growth period; GD_*r*_, rapid growth period; GD_st_, stable growth period; *V*_max_, maximum growth rate; *V*_mean_, mean growth rate; *W*_max_, maximum growth increment. * and ** represent significant differences at *p* < 0.05 and *p* < 0.01, respectively.

In the study area, sexual clusters were found only in the large patch, and their leaf area and plant height were higher than those of the asexual clusters (Figure 8a,c). Specifically, the maximum growth increment of the sexual clusters for both traits was significantly more than double that of asexual clusters (*p* < 0.01) (Figure 9c,o, Table 4). Their average and maximum growth rates of leaf area were significantly higher (*p* < 0.01) (Figure 9a,b, Table 4) and the growth rate of plant height was slightly higher than that of the asexual clusters (Figure 9m,n, Table 4). Compared to asexual clusters, the data when sexual clusters began the rapid growth (*D*_*r*_), reached maximum growth rate (*D*_vmax_), began stable growth (*D*_st_) and terminated growth (*D*_*t*_) significantly earlier (*p* < 0.01) (Figure 9e–h, Table 4), resulting in a significantly shorter rapid growth period (GD_*r*_) and total growth period (GD) of leaf area (*p* < 0.05) (Figure 9g,l, Table 4). In addition, the time when their plant height began rapid growth (*D*_r_), reached the maximum growth rate (*D*_vmax_), and began the stable growth (*D*_st_) was significantly later than that of the asexual clusters (*p* < 0.01) (Figure 9q–s, Table 4), making the slow and rapid growth periods (GD_sl_ and GD_*r*_) of plant height significantly longer than those of the latter (*p* < 0.01) (Figure 9u,v, Table 4).

**TABLE 4:** Growth characteristics comparison of leaf area and plant height between sexual and asexual ramets.

Trait	Growth parameters	Clusters		ANOVA	
		Sexual	Asexual	*F*	*p*
Leaf area	*V*_mean_ (cm^2^/day)	**5.8 ± 1.0**	**2.3 ± 1.0**	**47.83**	**< 0.001**
	*V*_max_ (cm^2^/day)	**14.8 ± 2.7**	**5.1 ± 2.8**	**48.64**	**< 0.001**
	*W*_max_ (cm^2^)	**602 ± 142**	**330 ± 134**	**15.42**	**0.001**
	*D*_*o*_ (DOY)	85 ± 6	84 ± 5	0.16	0.739
	*D*_*r*_ (DOY)	**116 ± 3**	**122 ± 5**	**6.85**	**0.005**
	*D*_vmax_ (DOY)	**129 ± 5**	**142 ± 9**	**10.86**	**0.019**
	*D*_st_ (DOY)	**141 ± 8**	**163 ± 15**	**10.00**	**0.006**
	*D*_*t*_ (DOY)	**180 ± 5**	**205 ± 17**	**12.80**	**0.003**
	GD_sl_ (day)	31 ± 7	37 ± 8	2.98	0.105
	GD_*r*_ (day)	**25 ± 6**	**41 ± 12**	**8.82**	**0.010**
	GD_st_ (day)	38 ± 6	42 ± 16	0.32	0.581
	GD (day)	**94 ± 9**	**120 ± 21**	**8.53**	**0.011**
Plant height	*V*_mean_ (cm/day)	1.1 ± 0.5	1.4 ± 0.2	1.71	0.211
	*V*_max_ (cm/day)	2.6 ± 0.7	2.7 ± 0.3	0.35	0.563
	*W*_max_ (cm)	**74 ± 12**	**125 ± 23**	**25.59**	**0.002**
	*D*_*o*_ (DOY)	107 ± 6	118 ± 4	3.40	0.118
	*D*_*r*_ (DOY)	**88 ± 1**	**86 ± 3**	**14.18**	**0.002**
	*D*_vmax_ (DOY)	**97 ± 3**	**103 ± 3**	**14.73**	**0.002**
	*D*_st_ (DOY)	**118 ± 9**	**133 ± 6**	**15.45**	**0.001**
	*D*_*t*_ (DOY)	167 ± 31	175 ± 5	0.83	0.382
	GD_sl_ (day)	**9 ± 3**	**17 ± 3**	**24.81**	**0.000**
	GD_*r*_ (day)	**20 ± 6**	**31 ± 4**	**13.61**	**0.002**
	GD_st_ (day)	49 ± 25	42 ± 4	0.80	0.390
	GD (day)	79 ± 31	90 ± 7	1.27	0.283

*Note:* Values are presented as mean ± SD. Differences between the asexual and sexual clusters were analyzed using ANOVA. The abbreviations for each parameter are the same as those listed in Table 2. Bold font is used to designate parameters for which statistically significant differences were observed.

### 3.4 Changes in Trait Pair Ratios and Allometric Characteristics During Clonal Expansion

**FIGURE 10:**
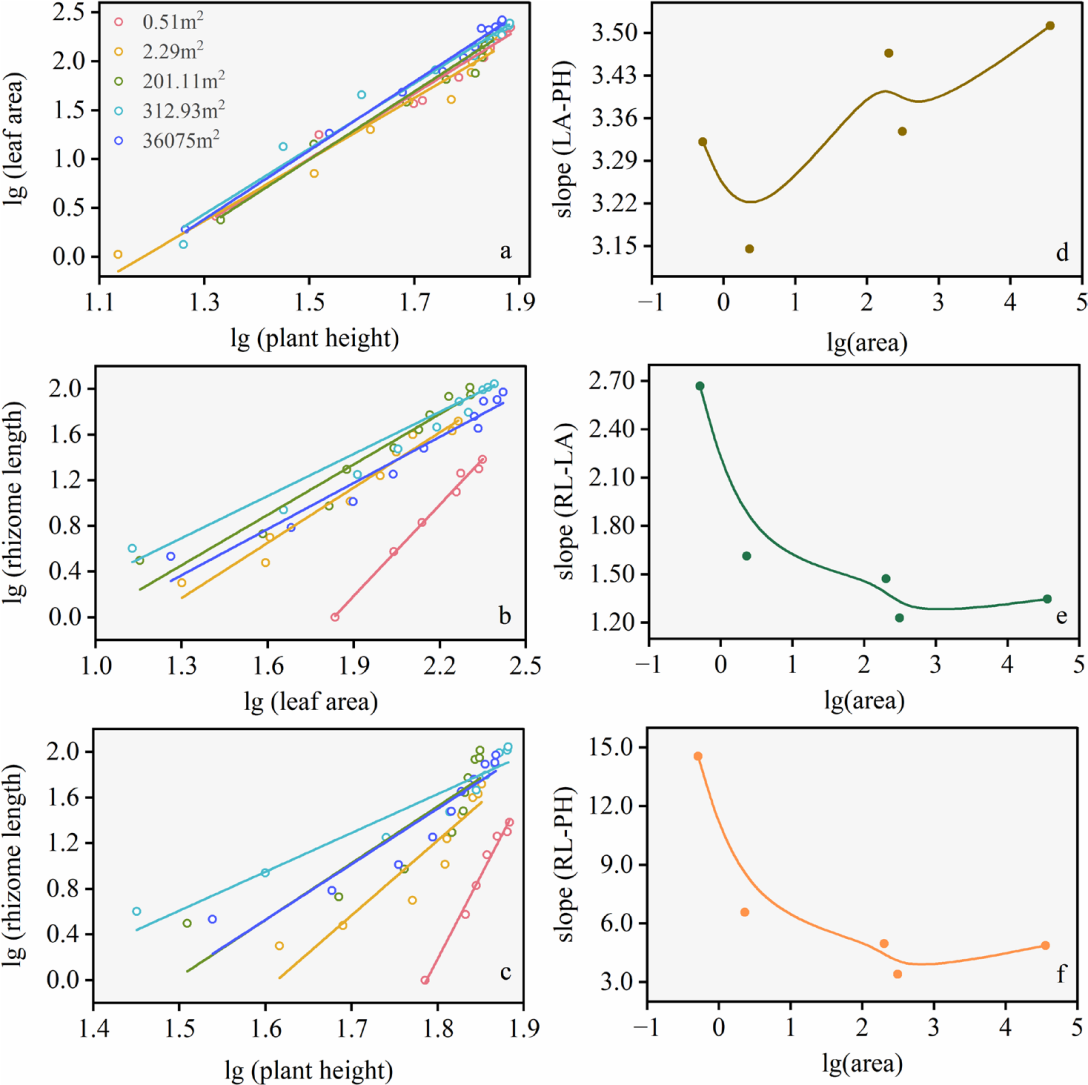
The allometric growth curve of leaf area to plant height (LA‐PH, a), rhizome length to leaf area (RL‐LA, b), rhizome length to plant height (RL‐PH, c) of different patch sizes and their slope changes during patch expansion (d, e). The allometric growth model (*y* = *ax* ^*b*^) was applied, mean trait values per patch were log‐transformed (lg *y* = lg *a* + *b* lg *x*), followed by Standardized Major Axis regression (SMA) to establish growth relationships for trait pairs. Field observations revealed that aboveground plant height and leaf area of *P. villosa* stabilized after mid‐July, whereas rhizome length continued increasing until early November, suggesting stage‐specific resource allocation. Consequently, trait growth relationships were only conducted for the period prior to mid‐July.

During the early growing season, all trait pairs across patches exhibited significant allometric growth patterns deviating from isometry (slope ≠ 1, Table 5). Specifically, positive allometric relationships (slope > 1) were detected for leaf area (LA) versus plant height (PH) of asexual clusters, rhizome length (RL) versus asexual cluster LA, and RL versus asexual cluster PH (Table 5, Figure 10a–c), indicating faster growth in the former traits. The allometric slope of LA relative to PH generally showed an increasing trend, with the largest patch having the highest value, while the small patch was lower than the other patches (Figure 10d). The relationships between belowground (RL) and aboveground traits (LA and PH) displayed stage‐dependent characteristics: during clonal expansion from micro‐ to medium‐sized patches, the standardized major axis (SMA) slopes of RL relative to asexual cluster PH and LA decreased obviously with clonal expansion, whereas these slopes increased during sexual reproduction expansion from medium‐ to large‐sized patches (Table 5, Figure 10e,f). Moreover, the micro‐patch exhibited steeper SMA slopes for RL‐PH and RL‐LA than other patches, and the larger medium‐sized patch (312.93 m^2^) exhibited lower SMA slopes for RL‐LA and RL‐PH than the small patch, and lower RL‐PH slopes than the larger patch (Figure 10e,f).

**TABLE 5:** Summary statistics and significance from Standardized Major Axis regression (SMA).

Trait pair	Patch area	Slope	Intercept	*R* ^2^	*p*	*R* ^2^_1_	*p*_1_
LA‐PH	0.51 m^2^	3.321	−3.976	0.973	**< 0.001**	0.994	**< 0.001**
	2.29 m^2^	3.145	−3.720	0.962	**< 0.001**	0.991	**< 0.001**
	201.11 m^2^	3.467	−4.218	0.970	**< 0.001**	0.994	**< 0.001**
	312.93 m^2^	3.338	−3.900	0.973	**< 0.001**	0.973	**< 0.001**
	36,075 m^2^	3.512	−4.179	0.992	**< 0.001**	0.999	**< 0.001**
RL‐LA	0.51 m^2^	2.668	−4.883	0.993	**< 0.001**	0.997	**< 0.001**
	2.29 m^2^	1.613	−1.928	0.963	**< 0.001**	0.933	**< 0.001**
	201.11 m^2^	1.472	−1.457	0.937	**< 0.001**	0.845	0.002
	312.93 m^2^	1.230	−0.969	0.954	**< 0.001**	0.699	0.025
	36,075 m^2^	1.347	−1.382	0.945	**< 0.001**	0.790	0.007
RL‐PH	0.51 m^2^	14.540	−25.985	0.985	**< 0.001**	1.000	**< 0.001**
	2.29 m^2^	6.563	−10.589	0.865	**< 0.001**	0.994	**< 0.001**
	201.11 m^2^	4.973	−7.428	0.776	**< 0.001**	0.981	**< 0.001**
	312.93 m^2^	3.404	−4.496	0.929	**< 0.001**	0.986	**< 0.001**
	36,075 m^2^	4.871	−7.264	0.882	**< 0.001**	0.989	**< 0.001**

*Note: R* ^2^_1_ and *p*_1_ are the statistics and significance that the specific SMA slope is different from 1.

Abbreviations: LA‐PH, the allometric growth curve of leaf area to plant height; RL‐LA, rhizome length to leaf area; RL‐PH, rhizome length to plant height. Bold font is used to designate parameters for which statistically significant differences were observed.

## 4 Discussion

### 4.1 Differences in the Growth Characteristics Among Morphological Traits

The growth of different morphological traits of *P. villosa* is notably asynchronous, which was particularly evident in the growth of the aboveground and belowground parts. Aboveground traits, including plant height and leaf area, completed their primary increases early in the growing season, with plant height first entering and completing the rapid growth period, followed by leaf area. This growth sequence assists *P. villosa* in optimizing vertical leaf positioning to enhance light capture and photosynthetic efficiency (Falster and Westoby 2003; Xiao et al. 2007). Unlike the aboveground parts, the growth period of belowground rhizomes lasted for approximately 8 months, demonstrating a robust expansive ability with an annual growth increment exceeding 200 cm. This characteristic enables *P. villosa* to possess well‐developed rhizomes with lengths more than 20 m and form a wide root network by generating adventitious roots from rhizome internodes (Chen et al. 2001; Zhong et al. 2024). This not only stabilizes *P. villosa* in windy environments but also enhances resource acquisition. Furthermore, the delayed rapid growth of rhizomes until post‐canopy development (Figure 4, Table 2) aligns with carbon‐partitioning trade‐offs observed in clonal plants (Radville et al. 2016; Yang et al. 2023), demonstrating how temporal segregation optimizes resource investment in sand dunes.

The growth patterns of the above‐ and below‐ground parts also exhibited distinct differences. Both leaf area and plant height displayed rapid unimodal growth, followed by a flat change, whereas the growth of rhizomes was characterized by a bimodal growth pattern, with continuous growth over extended periods. This divergence may be attributed to the different functions of these parts. Leaves are the primary sites for organic matter production, of which the spatial position affects the light capture of the plant (Givnish 1982; Liu et al. 2021), which need rapid growth to reach optimal positions and maintain a certain area for efficient photosynthesis. In contrast, rhizomes, as clonal reproductive organs, are closely related to plant foraging behavior and need to continuously expand over a long period to increase the range of resource acquisition (Lotscher 2006), particularly in environments where resources are limited and heterogeneously distributed (Yang et al. 2023). In addition, the July–September growth decline coincided with peak soil temperatures (exceeding 30°C for more than 50% of the time; Figure 1), suggesting thermal constraints. Notably, the soil temperature of the two rapid growth periods of rhizome was between 16°C and 29°C with an average of 24°C, which might be the suitable growth temperature for the rhizome of *P. villosa* (Figures 1 and 4). This temperature‐mediated growth plasticity, combined with functional specialization in spatial expansion and resource integration, ultimately enables *P. villosa* to balance ephemeral aboveground gains with persistent belowground investments in mobile dunes.

### 4.2 The Changes in Resource Allocation Strategies During the Expansion of Clonal Patches

In environments where resources are limited, clonal plants can respond to external stresses by adaptively adjusting traits—such as individual size, leaf area, and the expansion of clonal organs—to ensure their survival (Picotte et al. 2007; Wright et al. 2001; Yang et al. 2023). This study focused on the mobile sand dunes with extremely barren soil and heterogeneous distribution of water and nutrient resources (Zhang and Shao 2013). In this environment, the morphological traits of the asexual clusters of *P. villosa* exhibited different plasticity during the clonal expansion from micro to medium‐sized patches: plant height remained relatively stable, leaf area (per ramet) decreased in growth increment with an earlier termination (*D*_*t*_) and shorter total growth period (GD), while rhizome length increased in growth rate and increment with prolonged GD. These differences in plasticity reflect functional specialization and trade‐offs among traits, directly supporting H1's prediction of differential growth responses between photosynthetic and clonal traits as patch size increases. The low plant density of *P. villosa* patches (0.17–5.88 ramets/m^2^, H. Ren, unpublished data, Figure 2) in the study area's mobile dune environment diminishes light competition (Liu et al. 2021; Xiao et al. 2007). Consequently, plant height functions mainly in structural maintenance and sand burial avoidance, demonstrating conservatism shaped by genetic and environmental adaptation, which is consistent with another study (Zhong et al. 2024). Contrasting with conservative plant height, leaf area (governing photosynthetic capacity) and rhizome length (indicating resource acquisition and clonal expansion) exhibited significant plastic trade‐offs. Among patches without sexual reproduction (micro‐ to medium‐sized patches), rhizome extension is the main sink of carbon consumption (Comas et al. 2005). As a rhizomatous species, *P. villosa* physiologically integrates resources (water, nutrients, photosynthates) across ramets in extreme environments (Yu et al. 2008, 2004), enabling larger patches to access broader resources. Patch expansion increases ramet abundance and spatial occupancy, enhancing growth‐limiting resources (e.g., water) availability via integration (Alamusa et al. 2017; Wang et al. 2015; Zhang et al. 2024). During initial colonization, micro‐patches face harsher conditions that increase rhizome extension costs, necessitating larger ramet leaf area to maximize photosynthetic support for establishment. While within larger patches, enhanced environmental stability and higher ramet abundance drive concurrent multi‐branch rhizome extension, thereby increasing photosynthate allocation demands. By reducing individual leaf area while maintaining photosynthetic capacity, *P. villosa* reallocates resources to rhizomes, ensuring resource acquisition and clonal reproduction. This coordinated above‐belowground adjustment in resource and temporal allocation ensures clonal expansion while maintaining photosynthetic output, representing a key strategy enabling *P. villosa* to transition from settlement to expansion in mobile dunes.

The adjustment of resource allocation between different plant parts is also an important strategy for plants to cope with external stress (Freschet et al. 2018; Schenk and Jackson 2002). Cloning traits, such as the lateral spread of rhizomes, have been found to be positively correlated with the size of above‐ground traits (Chelli et al. 2024; Klimesova et al. 2015), suggesting that clonality may play a role in influencing covariance among above‐ and below‐ground investments. In this study, the leaf area of asexual ramets exhibited positive allometric growth relative to plant height (SMA slope > 1), suggesting that *P. villosa* allocates more resources to leaf area expansion for enhanced photosynthetic efficiency. During clonal expansion, the ratios of rhizome length to leaf area and to plant height both exhibited progressive increases with patch enlargement. Meanwhile, rhizome length demonstrated positive allometry relative to both leaf area (RL‐LA slope: 1.230–2.668) and plant height (RL‐PH slope: 3.404–14.540) in the early growing season (a phase dominated by aboveground biomass accumulation). This strategic growth pattern reflects *P. villosa*'s adaptation as a rhizomatous perennial: since its aerial organs senesce annually, a greater proportion of photosynthates is allocated to rhizomes—structures that simultaneously facilitate clonal propagation and serve as carbon reservoirs. Notably, micro‐patches exhibited the highest relative growth rate (allometric index) of rhizomes to aboveground traits, yet the lowest relative growth accumulation. As clonal expansion progressed, this relative growth rate declined while the ratio of rhizome length to leaf area increased—a shift driven primarily by enhanced rhizome growth concomitant with reduced leaf area production, supporting H1's prediction of differential resource allocation trade‐offs between photosynthetic and clonal traits during clonal expansion. During initial colonization, micro‐patches feature higher plant density (5.88 clusters/m^2^, H. Ren, unpublished data), intense competition, and exclusively edge‐exposed positions, subjecting plants to severe abiotic stress. Although the relative growth rate (allometric index b > 1) of rhizome length to leaf area is elevated here, the harsh environment simultaneously reduces rhizome extension efficiency and necessitates greater resource allocation to photosynthetic organs to offset losses. Consequently, rhizome accumulation relative to leaf area remains constrained. As patches expanded, improved environmental stability, resource availability, and reduced competition (plant density 0.17–2.52 clusters/m^2^, H. Ren, unpublished data) enable more efficient resource integration by rhizomes. Despite the decline in relative growth rate, enhanced survival rates, sustained accumulation, and reduced leaf area collectively facilitate substantially greater cumulative rhizome growth. Therefore, this dichotomy between growth rate dynamics and cumulative biomass allocation reflects the combined effects of temporal integration and environmental constraints shaping *P. villosa*'s survival strategy in dynamic dune environments.

### 4.3 The Sexual Allocation Strategies During the Expansion of Clonal Patches

Sexual reproduction is an energy‐intensive process that requires significant resource support (Wang et al. 2018). Similar to previous studies, sexual clusters were relatively taller with larger leaves than asexual clusters (Givnish 1982), which may be an adaptation to meet the resource demands of the sexual reproduction process. According to the results of this study, sexual clusters not only remained in the flowering and fruiting phases for a longer period, but also grew taller and larger leaves within a shorter timeframe, indicating that the consumption of resources for sexual reproduction is both large and rapid. The higher LA/PH increment ratio and allometric slope observed in large, sexually reproducing patches are consistent with an increased requirement for photosynthetic production. However, the energy produced by sexual clusters alone may be insufficient. Relative to patches without sexual reproduction, the large patch showed a higher growth increment and resource allocation in above‐ground traits of asexual ramets, whereas rhizome growth and allocation decreased obviously. This suggests that *P. villosa* simultaneously allocated more resources to the photosynthetic organs of its asexual ramets, boosting photosynthate production to support sexual reproduction. However, transitioning from medium‐sized patches (asexual reproduction only) to large patches (including sexual reproduction), the positive allometric relationship of rhizomes relative to aboveground ramet traits re‐intensified (the allometric index increases). This indicated that within large patches, although *P. villosa* adaptively reduces rhizome growth, it still requires an elevated relative rhizome growth rate to ensure resource supply for sexual reproduction. In addition, sexual ramets wither shortly after fruiting, while rhizomes continue to grow, indicating that after the differentiation of flower spikes, sexual clusters are dedicated to sexual reproduction and cannot supply carbon for the expansion of rhizomes. The photosynthetic products of asexual clusters need to be used for both sexual reproduction and rhizome growth, which may also be the reason for the lower expansion of rhizomes in these plots compared to the others. This also implies that *P. villosa* sacrifices the carbon used for rhizome growth to support sexual reproduction, supporting H2's prediction of distinct allocation patterns compared to smaller, solely vegetative patches. This further indicates that under the capability of resource integration provided by rhizomes, rhizomatous plants are able to regulate the overall resource allocation patterns within patches (Liu et al. 2016; Yang et al. 2023), which may make their reproductive strategies in such environments depend on the availability of resources and the size of the patches.

In a study on the grassland ecosystem of the rhizomatous plant *Leymus chinensis*, sexual ramets were found in smaller patches of the plant (Zhou et al. 2015). However, in this study, sexual ramets of *P. villosa* were only found in the large patch of 36,075 m^2^ (lg area > 4), while the patches before engaging in sexual reproduction focused on rhizome expansion in arid and sandy environments. We also found that the ratio of belowground to aboveground growth and their allometric relationship both shifted between medium and large patches (Figure 6), suggesting that *P. villosa* likely initiates preparation for strategic adjustment from clonal to sexual reproduction in mobile dunes when patch size reaches a lg‐transformed area of 2–3. This confirms H2's prediction of a critical patch size threshold enabling sexual reproduction, and indicates that rhizomatous plants with long lifespans even up to 50 years (de Witte and Stöcklin 2010) may extend the process of “seed‐ramet‐sexual reproduction‐new seed” and allocation strategies adjustment over a longer cycle in extreme environments, which is unlike annual and perennial plants in more suitable conditions that adjust resource allocation strategies within a short period of 1–2 years. This approach is similar to that of individual plants (such as *Meconopsis*) in extreme environments which accumulate resources over long periods to support sexual reproduction (Kelly et al. 2021). However, individual plants can only acquire more resources by increasing their root biomass and range, while rhizomatous plants can expand their rhizome systems and increase the occupied space of their patches, which may also benefit the allocation of sexual reproductive resources with the help of clonal integration (Demetrio and Coelho 2023; Dong et al. 2023). Furthermore, research has shown that *P. villosa* commonly employs clonal reproduction over large areas, with a relatively uniform genetic makeup (Chen et al. 2001; Wang et al. 1999). This study consistently found that only large patches exhibited sexual reproduction, suggesting that *P. villosa* patches require significant temporal and spatial expansion before they can secure sufficient resources to support sexual reproduction in extreme environments, which may limit the natural persistence capability of vegetation predominantly composed of rhizomatous plants. Consequently, further investigation is warranted to understand how the threshold of the growth period and size of patches that can support sexual reproduction differ across a broader distribution range and varying abundances of habitat resources, which will enhance our understanding of the application and management of clonal plants in sand fixation and desertification control. In addition, although this study lacked replicates across patch sizes and did not analyze the effects of sand burial depth on rhizome growth traits, our investigation of key above‐ and below‐ground morphological traits across patches spanning an exceptionally broad size gradient—combined with rigorous data collection and analysis—provides preliminary insights into shifts in resource allocation patterns during *P. villosa*'s expansion from micro‐ to large‐sized patches and its transition from clonal to sexual reproductive strategies. Future research should increase patch replicates, investigate rhizome depth distribution, and assess more direct resource allocation traits (e.g., biomass, bud bank ratio) to deepen understanding.

## 5 Conclusions

In arid sandy habitats, the rhizomatous clonal herb *P. villosa* exhibits significant asynchrony in the peak growth among its primary morphological traits. Aboveground traits (plant height, leaf area) display rapid unimodal growth early in the season, while belowground rhizomes exhibit prolonged bimodal growth spanning about 8 months, reflecting temporal niche partitioning to optimize carbon investment. During clonal expansion, asexual ramets show divergent plasticity. Plant height remains conservative, leaf area per ramet decreases with reduced growth duration, while rhizome length increases markedly in growth rate and increment as patches expand from micro to medium sizes. This shift signifies a trade‐off from photosynthetic investment (small patches) toward clonal expansion (medium patches), enabled by physiological integration that enhances resource acquisition in larger patches. After clonal patches reach a certain size (lg‐transformed area of 2–3), the growth allocation of the aboveground parts relative to the rhizome increased to prepare the population for sexual reproduction. However, due to the energy‐intensive nature of sexual reproduction and the resource accumulation method through patch expansion, sexual reproduction occurs exclusively in large patches (lg‐transformed area is more than 4) at the expense of underground rhizome growth, highlighting how *P. villosa* prioritizes spatial occupation via rhizomes and adjusts resource reallocation before committing resources to costly sexual propagation in resource‐limited dunes. Our findings underscore that rhizomatous plants adapt to extreme environments through stage‐specific adjustments in trait growth, phenology, and allometric allocation during clonal expansion. Future studies should quantify the thresholds of patch size and age enabling sexual reproduction across environmental gradients to optimize the use of clonal grasses in desertification control.

## Author Contributions

**Heng Ren:** conceptualization (equal), funding acquisition (lead), investigation (lead), methodology (lead), writing – original draft (equal), writing – review and editing (equal). **Wen Wang:** conceptualization (equal), data curation (equal), methodology (equal), visualization (lead), writing – original draft (lead), writing – review and editing (lead). **Wenzhi Zhao:** conceptualization (equal), methodology (equal), validation (equal), writing – review and editing (equal). **Zhibin He:** conceptualization (equal), methodology (equal), writing – review and editing (equal). **Jun Du:** conceptualization (equal), methodology (equal), writing – review and editing (equal).

## Ethics Statement

The authors have nothing to report.

## Conflicts of Interest

The authors declare no conflicts of interest.

## Supporting information


**Data S1:** ece372432‐sup‐0001‐DataS1.xlsx.

## Data Availability

The datasets analyzed during the current study were provided as Supporting Information.
